# Determining factors associated with the prevalence of knowledge, attitude, and practice in seeking skilled maternal healthcare services among women in a remote area of Gesha district

**DOI:** 10.1186/s12913-022-08710-y

**Published:** 2022-11-04

**Authors:** Sali Suleman Hassen, Mesfin Esayas Lelisho

**Affiliations:** Department of Statistics, College of Natural and Computational Science, Mizan-Tepi University, Tepi, Ethiopia

**Keywords:** Knowledge, Attitude, Practice, Skilled maternal care, ANC, DC, PNC

## Abstract

**Background:**

Skilled health care is essential for the mother's and newborn's health and well-being during pregnancy, labor, and the postpartum period. This study aimed to analyze women's knowledge, attitudes, and practices while requesting competent assistance for maternity healthcare in Gesha District, Southwest Region of Ethiopia.

**Methods:**

A community-based cross-sectional study design was conducted from September 20, 2021 to October 19, 2021. A total of 424 mothers participated in this study and a systematic sampling technique was used to select the respondents. The data were collected using a pretested and structured questionnaire. Statistical software SPSS-20 and R-4.1.2 were used to enter and analyze the data respectively. The factors associated with the prevalence of Knowledge, Attitude, and Practice in seeking Skilled Maternal Healthcare Services were identified using descriptive analysis and a binary logistic regression model.

**Results:**

This study result revealed that the overall proportions of good knowledge, positive attitude, and good practice in seeking skilled maternal health care services were 39.15%, 37.5%, and 34.67% respectively. Estimated odds of having knowledge, attitude, and practice were as follows: for having age between 20–24 years at first pregnancy 1.859, 1.86, and 1.799; having a plan for pregnancy 2.74,2.315 and 2.579; mothers attended elementary education 2.337, 2.565 and 3.312; having maternal age 20–24 years 4.336,4.989 and 5.870; maternal age 25–29 years 2.917, 3.794 and 4.017; maternal age 35–49 years 2.837, 2.991 and 3.412; having husbands who had attended elementary education level 2.736, 2.542 and 2.134; secondary and above education 3.464, 3.360 and 2.508; rich mothers 2.261, 1.995 and 2.452; having antenatal care 4 times and above 2.606, 2.570, 2.682; having transportation access 1.921, 1.956 and 2.404; having media access 1.979, 2.171 and 2.715 respectively. The odds of having attitude and practice respectively were as follows: married 1.762, and 2.208; having medium wealth index 1.933 and 2.424. The odds of having previous pregnancy complications was 2.147 which significantly affect the practice of seeking skilled maternal care assistance.

**Conclusions:**

This study discovered that the study participants' knowledge, attitude, and practice of skilled maternal health care are low. Associated factors included age at first pregnancy, planned pregnancy, maternal education level, husband’s education level, maternal age, antenatal care service visits, transport access, and access to media were found to significantly affect the knowledge, attitude, and practice of the respondents in seeking skilled maternal care assistance in common. The household wealth index was also associated with attitude. Pregnancy complications, current marital status, and household wealth index also significantly affect the practice of seeking skilled maternal care assistance. As a result of the findings, initiatives to increase women's knowledge, attitudes, and use of expert maternal health services in the research area are needed for women residing in rural areas.

## Background

The term "skilled maternal health services" refers to the medical care given to women before, during, and after childbirth which is given by a health professional with midwifery skills that can be provided at different levels (home, health centers, hospitals, and private sectors) [1, 2]. Access to skilled maternal health care services during pregnancy, labor, and the postpartum period is critical to the mother's and newborn's health and well-being. Maternal mortality and morbidity, on the other hand, continue to be major concerns [2]. Approximately 303,000 mothers died worldwide as a result of maternal causes during pregnancy, childbirth, and postpartum [3]. The developing world accounted for 99 percent (302,000) of maternal deaths worldwide, with Sub-Saharan Africa accounting for two-thirds (201,000) [4]. Ethiopia is one of the countries in Sub-Saharan Africa with one of the worst maternal death rates (MMR). MMR is 420 per 100,000 live births, according to the Ethiopian demographic health survey (EDHS) 2016 report [5].

Sustainable development goal (SDG) 3.1 aims to reduce maternal mortality to under 70 deaths per 100,000 live births by 2030 [6]. Only half of women in poor countries, on the other hand, get the healthcare they require. The Ethiopian government has made significant headway in lowering maternal death rates. According to the Ethiopian Demographic Health Survey, the maternal death rate has decreased from 676 per 100,000 live births in 2011 to 412 in 2016 [7]. Despite advancements in maternal healthcare, considerable barriers to access and low usage of maternal health services still exist. Only 62 percent, 28 percent, and 17 percent of Ethiopian women receiving competent antenatal care, skilled birth attendants, and postnatal care, respectively, received skilled antenatal care, skilled birth attendants, and postnatal care [7]. Maternal healthcare services are clearly the most critical interventions for preventing mother morbidity and mortality, yet simply having access to care is insufficient to enhance maternal health outcomes. In low- and middle-income nations, poor infrastructure, poor quality care, and inequality significantly hinder efforts to expand maternity services [8].

Maternal education and awareness of trained providers are constant determinants of prenatal care at the individual level [8–13]. Unplanned pregnancies and women who gave birth multiple times (multiparous) were less likely to seek antenatal care [14]. At the structural level, a lack of essential infrastructures such as transportation and telecommunications networks hampered access to antenatal care services [15–17]. Past research in Sub-Saharan Africa has found that education, primiparous women, previous antenatal care visits, and awareness of trained providers are all important predictors of skillful delivery [8–12, 18–21]. Other research, on the other hand, shows that multiparous has a favorable impact on institutional delivery [22, 23]. Furthermore, evidence suggests that antenatal care attendance, desired pregnancy, and birth difficulties are all important predictors of postnatal care service use [20, 24–26].

The Ethiopian government intends to reduce maternal mortality, infant mortality, and morbidity by strengthening maternal healthcare system interventions, which include increasing birth attendants, meeting unmet family planning needs, improving childbirth care quality, and increasing health system financing, but maternal mortality remains an unfinished issue that requires further investigation [27]. Even though several types of research have been undertaken in Ethiopia on the consumption of maternal health services [9–11, 18, 21, 28], few have addressed the women's level of awareness and attitude toward skilled maternity health care. As a result, the purpose of this study was to evaluate women's knowledge, attitude, and practice of skilled maternity care, as well as the factors that influence them, in a remote area in Gesha district, South West Regional State.

### Description of the study area

Gesha is a district in the South West Region of Ethiopia. Part of the Keffa Zone, Gesha is bordered on the south by Bita, on the west by the Sheka Zone, on the north by the Oromia Region and Sayilem, and on the east by Gewata. Towns in Gesha include Deka. The northern part of Gesha was separated to create Sayilem district, the eastern part was added to Gewata district, and the southern part to Bita district. Based on the 2007 Census conducted by the CSA, this district has a total population of 85,104, of whom 41,441 are men and 43,663 women; 81,671 or 96% of its population are rural dwellers. The majority of the inhabitants were Protestants, with 44.62% of the population reporting that belief, 41.02% practicing Ethiopian Orthodox Christianity, and 13.25% of the population were Muslim.

### Study design and setting

A community-based cross-sectional study was conducted with a quantitative data collection technique to assess knowledge, attitude, and practice on seeking skill assistance for maternal healthcare services and associated factors among women in rural parts of Gesha woreda. The actual data collection process was carried out from September 20, 2021 to October 19, 2021 (a one-month survey). All women who gave live birth or stillbirth in the last 2 years in rural parts of Gesha woreda were the study participants. The analytical Method of data analysis was used to combine the power of the scientific methods with the use of formal processes to solve the problems related to seeking skill assistance for maternal healthcare services.

### Eligibility criteria

Women between 15–49 years of age, women with 2 years of post-delivery or stillbirth, Women, who were mentally and physically capable of being interviewed, and Women who were permanent residents were included in the study.

Women who were critically ill, could not talk or listen, and women who were caregivers but not the actual mother of the baby were excluded from the study.

### The sample size determination

The required sample for this study was determined by using a single population proportion sample size calculation formula considering the following assumptions. 95% confidence interval (CI), 5% margin of error, and due to no prior studies done in the study area, the researcher took the maximum proportion which equals 50%.

$$\mathrm n=\frac{\left({\displaystyle{\mathrm Z}_{\textstyle\frac{\mathrm a}2}}\right)^{\mathit2}\;\mathrm{pq}}{\mathrm d^2}$$

α = 0.05 (level of significance) with 95% confidence interval.

P = estimated prevalence rate = 0.5

q = 1-p = 1–0.5 = 0.5

d = margin of error of 0.05

n = the sample size

Thus the sample size for this study was $\mathrm{n}=\frac{{[1.96]}^{2}0.5[0.5]}{{0.05}^{2}}=384.16\approx 385$ with Contingency for non-response and incompleteness = 10%.

Therefore, the final sample size for this study was $385+38.5=423.5\approx 424$

### Sampling procedures

Gesha woreda’s rural parts were selected purposely to find out factors related to maternal health care service and due to the accessibility of the service might be low compared to the counterparts who were from urban areas. For selecting respondents from the sampling frame of 424 eligible households, a systematic sampling technique was used. Systematic Sampling is a type of probability sampling method where random starting points with fixed intervals are used to select members from a larger population. The household in the direction of the tip of the pen was started with a nearby first. After a successful interview of each household, the interviewer continued to the immediate nearby household of the eligible woman until the required sample size was achieved in the study area. When two eligible women existed in one household, the one with recent birth was selected.

### Measuring instruments

A questionnaire was adopted from previous similar studies [29] and also partly developed from various literature reviews that could address the objectives of the study [30]. The questions and statements were grouped and arranged according to the particular that they can address. After extensive revision, the final version of the English questionnaire was developed. An individual who was a graduate of English and knows the local language which is called “kafinoonoo” translated the English version to “kafinoonoo”. Another individual of similar ability then re-translated the final or the agreed “kafinoonoo” version of the questionnaire back to the English with the first to check for any inconsistencies or distortion in the meaning of words in the content of the instrument.

### Operational definitions

Skilled assistance: refers to maternity services (antenatal care, Delivery care, and postnatal care) by a health professional with midwifery skills that can be provided at different levels (home, health centers, hospitals, private sector) by a skilled care provider [1, 31].

Skilled care providers: are health professionals (midwives, nurses, and doctors) who have been effectively educated and trained in the skills needed to manage normal (uncomplicated) pregnancies as well as in the identification, management, or referral of complications [1, 31].

Non-skilled providers include health extension workers (HEWs), traditional birth attendants (TBAs), and relatives or family members who cannot fulfill the definition of a skilled provider [1, 31].

Good knowledge and poor knowledge: Women who scored above the mean on knowledge assessment questions were judged to have good knowledge, while those who scored below the mean were considered to have poor knowledge about skilled maternal health services [30].

Positive and negative attitude: Attitude was measured by using the Likert scale (1 = strongly agree, 2 = agree, 3 = disagree, and 4 = strongly disagree). Participants with a positive attitude scored above the mean on the attitude evaluation questions, whereas those with a negative attitude scored below the mean. Practice (antenatal care, skilled delivery, and postnatal care utilization) was measured such that participants who respond above the mean of the practice assessment question were considered as having a good practice, and if below the mean they were considered as having poor practice [30].

Antenatal care (ANC): is a maternal healthcare service provided by skilled healthcare professionals to pregnant women which are provided throughout pregnancy to ensure the best health outcomes for both the mother and the newborn [32].

Postnatal care (PNC): is the care given to the mother and her newborn baby immediately after the birth and for the first six weeks of life [33]

### Study variables

The outcome variables for this study were knowledge, attitude, and practice each of which has two categories (dichotomous). The outcome variables are denoted as ${\mathrm{Y}}_{\mathrm{i}}, {\mathrm{w}}_{\mathrm{i}},\mathrm{ and }{\mathrm{Z}}_{\mathrm{i}}$ for knowledge, attitude, and practice respectively.

$${\mathrm Y}_{\mathrm i}=\left\{\begin{array}{c}1,\;\mathrm{good}\;\mathrm{knowledge}\\0,\;\mathrm{poor}\;\mathrm{knowledge}\end{array}\right.$$

$${\mathrm W}_{\mathrm i}=\left\{\begin{array}{c}1\;,\mathrm{positive}\;\mathrm{attitude}\\0,\;\mathrm{negative}\;\mathrm{attitude}\end{array}\right.$$

$${\mathrm Z}_{\mathrm i}=\left\{\begin{array}{c}1,\;\mathrm{good}\;\mathrm{practice}\\0,\;\mathrm{poor}\;\mathrm{practice}\end{array}\right.$$

where, $\mathrm{i}=\mathrm{1,2},3\dots . 424$ which indicates the ${\mathrm{i}}^{\mathrm{th}}$ individual mother.

Explanatory variables for this study were Socio-demographic variables (maternal age, mother’s education level, marital status, husband’s education level, household wealth index, women occupation, and the number of children ever born); obstetric variables(age at first pregnancy, plan about the last pregnancy, previous pregnancy complication, and antenatal care follow up) and structural variables(transportation access and media access).

### Data processing and analysis

After validating the data, the investigator entered the data using SPSS version 20 and exported it to R version 4.1.2 statistical software packages for data cleaning and analysis. Computer frequencies and summary statistics were used to describe the study population by variables of interest. Any errors identified at this time were corrected after revision of the original data using the code numbers and statistical commands. By including all significant factors from the univariate analysis with a significance level of 25%, a multivariable logistic regression analysis was performed [34, 35]. The degree of association between independent and dependent variables was analyzed by a multivariable logistic regression model using binary analysis with 95% CI.

Binary logistic regression is often applicable in a situation where the dependent variable is dichotomous such as the presence or absence of a particular event and the explanatory variables are of any type. The Bernoulli distribution for Bernoulli trial specifies probabilities P (Y = 1) = π and P(Y = 0) = 1- π, for which E (Y) = π.

The general model for binary logistic regression is as follows:

1 $$\mathrm{logit}\left(\mathrm\pi\left({\mathrm x}_{\mathrm i}\right)\;\right)\;=\;\log\;\left(\frac{\mathrm\pi\left({\mathrm x}_{\mathrm i}\right)}{1-\mathrm\pi\left({\mathrm x}_{\mathrm i}\right)}\right)\;=\;{\mathrm\beta}_{0\;+\;}{\mathrm\beta}_{1\;}{\mathrm x}_1+{\mathrm\beta}_{2\;}{\mathrm x}_2\;+\cdot\cdot\cdot+{\mathrm\beta}_{\mathrm k}{\mathrm x}_{\mathrm k}$$

where: ${\mathrm{x}}_{\mathrm{i}}$ is an independent variable in the model, π: the probability of success, 1-π: the Probability of failure, ${\beta }_{o}$is constant terms, ${\beta }_{i}$is the coefficients/slope of the independent variable in the model.

### Parameter estimation

The maximum likelihood and Wald tests were used for parameter estimation methods in fitting the logistic regression model [36].

The maximum likelihood estimates of the parameters could be obtained by maximizing the log-likelihood function form which is given by:

2 $$\pi\left(x_i\right)\;=\;\frac{\exp\left(\mathit\;\beta_{\mathit0}\mathit\;+{\mathit\;\mathit\beta}_1X_{\mathit1}\mathit\;+\;{\mathit\;\mathit\beta}_{\mathit2}X_{\mathit2}\mathit\;+\;\cdot\cdot\cdot\cdot\;+\;{\mathit\;\beta}_KX_k\right)}{1\;+\;\exp\left(\;\beta_{\mathit0}\mathit\;+{\mathit\;\beta}_1X_1\mathit\;+\;{\mathit\;\beta}_{\mathit2}X_{\mathit2}\mathit\;+\;\cdot\cdot\cdot\cdot\;+\;{\mathit\;\beta}_KX_k\right)}$$

Since observing values of $\mathrm{Y}$ say, ${\mathrm{Y}}_{\mathrm{i}}\mathrm{^{\prime}}\mathrm{s }(\mathrm{i}=1, 2\dots \mathrm{n})$ are independently distributed as Bernoulli, the maximum likelihood function of Y is given by:

3 $$\mathrm L\left(\beta\;/\;y\right)\;=\;\prod\limits_{i=1}^n\;P\mathit\;\mathit{\left({y_{i\;}/\;x_i\;}\right)}\mathit\;=\;\prod\limits_{i=1}^n\left[\frac{{}_{\mathit e}x_{\mathit i}\beta_{\mathit i}}{1\;+\;{}_ex_{\mathit i}\beta_{\mathit i}}\right]^{y_{\mathit i}}\;=\;\left[\frac1{1\;+\;{}_{\mathit e}{{\mathit x}_{\mathit i}{\mathit\beta}_{\mathit i}}}\right]^{\left(1-y_{\mathit i}\right)}$$

### The Wald test statistic

The Wald test is a way of testing the significance of particular explanatory variables in a statistical model [37]. Wald χ^2 ^statistics are used to test the significance of individual coefficients in the model and are calculated as follows:

4 $$W=\left(\frac\beta{se\left(\beta\right)}\right)^2\sim{\chi^2}_{(1)}$$

where se (β) is the standard error of regression coefficient β. W assumes chi-square distribution with one degree of freedom.

### Model adequacy checking

The Hosmer and Lemeshow test is commonly used to test for assessing the goodness of fit of the model and allows for any number of explanatory variables [37]. The receiver operating characteristic curve has been considered a statistical tool to evaluate the performance of model adequacy and it is a common technique for judging the accuracy of the fitted binary logistic regression model [38].

## Results

The goal of this study was to determine the prevalence of knowledge, attitude and practice, and associated factors among reproductive-aged women who gave birth within the last 2 years from rural Gesha woreda. To determine the prevalence and determinants of knowledge, attitude, and practice, descriptive and inferential analyses were employed.

**Fig. 1:**
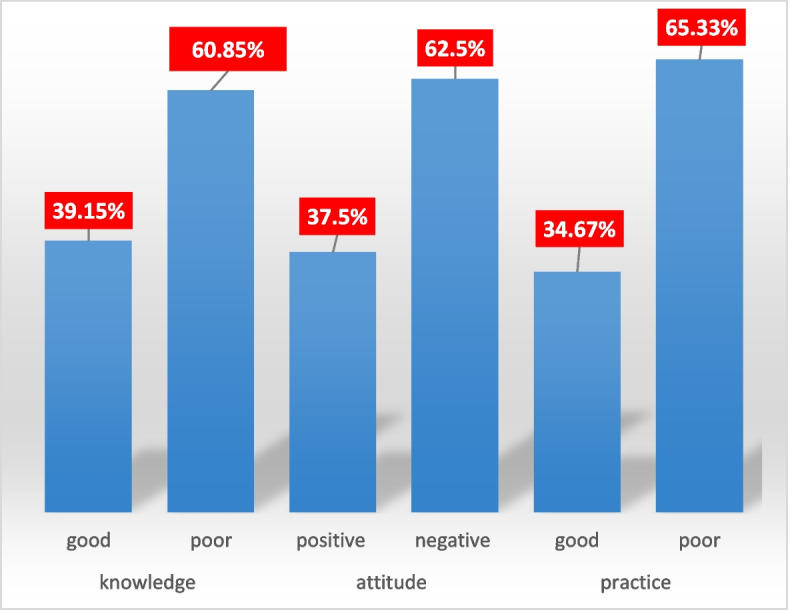
Prevalence of KAP on seeking skilled maternal healthcare services given in percentage

A total of 424 women participated in this study. The overall proportion of good knowledge, positive attitude, and good practice in seeking skilled maternal health care services were 39.15%, 37.5%, and 34.67% respectively with a 100% response rate (Fig. 1).

Among 130 women in the age group 15–19, 27(20.8%), 23(17.7%) and 19(14.6%) of them had good knowledge, positive attitude, and good practice in skilled assistance on maternal health services respectively. Of those mothers who were in the age group 35–49, 23(46.9%), 22(44.9%) and 21(42.9%) of them had good knowledge, positive attitude, and good practice toward skilled assistance in maternal care services respectively.

Among 196 mothers who had no formal education, above one-fourth (28.1%) had good knowledge, about one-fourth (25.5%) had a positive attitude and above one-fifth (21.4%) had good practice towards skilled assistance in maternal health services. Among 178 mothers who were never married, 55(30.9%), 51(28.7%), and about one-fourth 44 (24.7%) of them had good knowledge, positive attitude, and good practice respectively. Of those mothers whose husbands were in secondary and above education level, about half (48%), 35(46.7%), and 30(40%) of the mothers had good knowledge, positive attitude, and good practice of skilled assistance in maternal health service respectively. Regarding the household wealth index, only about one-third (32.9%) of poor mothers had good knowledge and above one-fourth (27.1%) of the respondents had good practice towards skilled assistance on maternal health services (Table 1).

**Table 1:** Socio-demographic characteristics regarding KAP on seeking skilled maternal healthcare services given in percentage

Variables	Categories	Knowledge		Attitude		Practice	
		Poor	Good	Negative	Positive	Poor	good
		Count (%)	Count (%)	Count (%)	Count (%)	Count (%)	Count (%)
Maternal age	15–19	103(79.2)	27(20.8)	107(82.3)	23(17.7)	111(85.4)	19(14.6)
	20–24	46(41.4)	65(58.6)	48(43.2)	63(56.8)	50(45.0)	61(55.0)
	25–29	35(50.7)	34(49.3)	35(50.7)	34(49.3)	38(55.1)	31(44.9)
	30–34	48(73.8)	17(26.2)	48(73.8)	17(26.2)	50(76.9)	15(23.1)
	35–49	26(53.1)	23(46.9)	27(55.1)	22(44.9)	28(57.1)	21(42.9)
Mother’s Education Level	No education	141(71.9)	55(28.1)	146(74.5)	50(25.5)	154(78.6)	42(21.4)
	Elementary	70(44.6)	87(55.4)	72(45.9)	85(54.1)	74(47.1)	83(52.9)
	High School and above	47(66.2)	24(33.8)	47(66.2)	24(33.8)	49(69.0)	22(31.0)
Marital Status of Mothers	Never Married	123(69.1)	55(30.9)	127(71.3)	51(28.7)	134(75.3)	44(24.7)
	Married/living together	100(56.2)	78(43.8)	101(56.7)	77(43.3)	103(57.9)	75(42.1)
	Divorced/separated/widowed	35(51.5)	33(48.5)	37(54.4)	31(45.6)	40(58.8)	28(41.2)
Husband’s education level	no education	126(75.9)	40(24.1)	127(76.5)	39(23.5)	128(77.1)	38(22.9)
	Elementary	93(50.8)	90(49.2)	98(53.6)	85(46.4)	104(56.8)	79(43.2)
	secondary and above education	39(52.0)	36(48.0)	40(53.6)	35(46.7)	45(60.0)	30(40.0)
Household Wealth Index	Poor	139(67.1)	68(32.9)	142(68.6)	65(31.4)	151(72.9)	56(27.1)
	Medium	77(54.6)	64(45.4)	78(55.3)	63(44.7)	80(56.7)	61(43.3)
	Rich	42(55.3)	34(44.7)	45(59.2)	31(40.8)	46(60.5)	30(39.5)
Women occupation	Farmer	30(78.9)	8(21.1)	30(78.9)	8(21.1)	31(81.6)	7(18.4)
	Housewife	47(54.7)	39(45.3)	50(58.1)	36(41.9)	52(60.5)	34(39.5)
	daily worker	66(55.5)	53(44.5)	69(58.0)	50(42.0)	71(59.7)	48(40.3)
	Merchant	66(64.1)	37(35.9)	67(65.0)	36(35.0)	71(68.9)	32(31.1)
	government employed	49(62.8)	29(37.2)	49(62.8)	29(37.2)	52(66.7)	26(33.3)
Number of Children ever born	1	82(59.9)	55(40.1)	85(62.0)	52(38.0)	88(64.2)	49(35.8)
	2–4	104(59.1)	72(40.9)	107(60.8)	69(39.2)	115(65.3)	61(34.7)
	> 4	72(64.9)	39(35.1)	73(65.8)	38(34.2)	74(66.7)	37(33.3)

Among 252 women whose first pregnancy was within the age group 15–19, 80(31.7%), 76(30.2), and 69(27.4) of them had good knowledge, positive attitude, and good practice toward skilled assistance in maternal care services respectively, like prenatal care and postnatal care. Of those mothers whose last pregnancy was not planned, above one-third (34.4%) of the respondents had good knowledge, about one-third (32.6%) had a positive attitude and only 29.8% of the respondents had a good practice. Regarding previous pregnancy complications, a total of 197 women had previous pregnancy complications and from which, 49.7%, 44.2%, and 43.7% of the women had good knowledge, positive attitude, and good attitude in seeking skilled assistance on maternal care services respectively. Among those mothers who attended the number of antenatal care service visits less than 4 times, only 29% had good knowledge, above one-fourth (27.4%) had a positive attitude and about one-fourth (24.7%) had good practice in seeking skilled assistance on maternal care service(Table 2).

**Table 2:** Obstetric-related characteristics regarding KAP

Variables	Categories	Knowledge		Attitude		Practice	
		Poor	Good	Negative	Positive	poor	Good
		Count (%)	Count (%)	Count (%)	Count (%)	Count (%)	Count (%)
Age at first pregnancy	15–19	172(68.3)	80(31.7)	176(69.8)	76(30.2)	183(72.6)	69(27.4)
	20–24	86(50.0)	86(50.0)	89(51.7)	83(48.3)	94(54.7)	78(45.3)
Plan about the last pregnancy	No	185(65.6)	97(34.4)	190(67.4)	92(32.6)	198(70.2)	84(29.8)
	Yes	73(51.4)	69(48.6)	75(52.8)	67(47.2)	79(55.6)	63(44.4)
Previous pregnancy complication	No	153(67.4)	74(32.6)	155(68.3)	72(31.7)	166(73.1)	61(26.9)
	yes	105(53.3)	92(46.7)	110(55.8)	87(44.2)	111(56.3)	86(43.7)
ANC follow up	< 4	184(71.0)	75(29.0)	188(72.6)	71(27.4)	195(75.3)	64(24.7)
	≥ 4	74(44.8)	91(55.2)	77(46.7)	88(53.3)	82(49.7)	83(50.3)

Among those mothers who had no transport access, about one-third (32.9%) had good knowledge, less than one-third (31.4%) had a positive attitude and above one-fourth (27.9) had a good practice. Regarding media access, among those mothers who had no media access, 74(29.2%) had good knowledge, 27.3% had a positive attitude, and about one-fourth (23.7%) had good practice in seeking skilled assistance on maternal care services (Table 3).

**Table 3:** Structural characteristics related to KAP in seeking skilled maternal healthcare services given in percentage

Variables	Categories	Knowledge		Attitude		Practice	
		Poor	Good	Negative	Positive	poor	Good
		Count (%)	Count (%)	Count (%)	Count (%)	Count (%)	Count (%)
Transportation access	No	173(67)	85(32.9)	177(68.6)	81(31.4)	186(72.1)	72(27.9)
	Yes	85(51.2)	81(48.8)	88(53.0)	78(47.0)	91(54.8)	75(45.2)
Media	No	179(70.8)	74(29.2)	184(72.7)	69(27.3)	193(76.3)	60(23.7)
	Yes	79(46.2)	92(53.8)	81(47.4)	90(52.6)	84(49.1)	87(50.9)

### Multivariable binary logistic regression results

Three separate binary logistic regression models were fitted for the three outcome variables such as knowledge, attitude, and practice. Explanatory variables which are significant at a 25% level of significance at a Univariable level were selected for multivariable binary logistic regression. A brief explanation of the results for each model was presented in the following sections.

### Factors associated with knowledge in rural parts of Gesha Woreda, Kaffa, Southwest Ethiopia

Based on the multivariable binary logistic analysis, age at first pregnancy, plan for pregnancy, maternal education, Maternal age, Husband’s educational level, Household Wealth index, ANC, Transport access, and Media access were found to be significant factors to know seeking skill assistance of maternal health care service while marital status, pregnancy complication, women occupation and number of children were not significant variables at 5% level of significance.

Mothers whose first pregnancy was at age interval 20–24 were 1.859 times [95% CI: 1.119–3.087] more likely than those mothers whose first pregnancy was at age groups 15–19 to know seeking skilled assistance of maternal health care service. When compared to respondents who had no plan for the pregnancy, those who had a plan were 2.274 times [95% CI: 1.348–3.835] more likely to know the skilled assistance of maternal health care services. Regarding maternal education, mothers who had elementary education were a 2.337 [95% CI: 1.347– 4.052] times higher likelihood of knowing skilled assistance on maternal health care services compared to those who had no formal education. The finding also revealed that mothers in the age group 20–24, 25–29, and 35–49 were 4.336 [95% CI: 2.220–8.472], 2.917[95% CI:1.377–6.179] and 2.835[95% CI:1.186–6.773] times more likely to know the skilled assistance respectively as compared to their counterparts who were in the age group 15–19.

Holding effect other covariates constant, the odds of knowing the skilled maternal assistance for those respondents whose husbands had elementary and secondary &above were 2.736 [95% CI: 1.545–4.846] and 3.464 [95% CI: 1.693–7.089] times greater than those whose husbands had no formal education respectively. Respondents who were in the medium household wealth index were 1.854 [95%CI: 1.048–3.281] times more likely to know skilled health assistance than those who were in the poor household wealth index. According to the findings of this study, respondents who attended the antenatal care service visits four times and above were 2.606 [95% CI: 1.573–4.318] times more likely to know seeking skilled assistance than those who had visited the ANC service less than 4 times. Knowledge was 1.921 [95%CI: 1.154–3.197] times more common among respondents who had transportation access compared with those mothers who had no transportation access. Mothers who had media access were 1.979[95%CI: 1.173–3.339] times more likely to know the skilled maternal care assistance than those who had no media access (Table 4).

**Table 4:** Responses of multivariable Binary logistic regression result for Knowledge

Variables	Categories	B	S.E	Sig	Exp(B)	95% C.I.for EXP(B)	
						Lower	Upper
Age at first pregnancy (ref: 15–19)	20–24	.620	.259	.017	1.859	1.119	3.087
Plan for the Pregnancy (ref: No)	Yes	.821	.267	.002	2.274	1.348	3.835
Maternal educational level (ref: No education)	Elementary	.849	.281	.003	2.337	1.347	4.052
	Secondary and above	.184	.358	.607	1.202	.596	2.425
Maternal age (ref:15–19)	20–24	1.467	.342	.000	4.336	2.220	8.472
	25–29	1.071	.383	.005	2.917	1.377	6.179
	30–34	.256	.411	.533	1.292	.578	2.889
	35–49	1.042	.444	.019	2.835	1.186	6.773
Husband’s educational level (ref: No education)	Elementary	1.007	.292	.001	2.736	1.545	4.846
	Secondary and above	1.242	.365	.001	3.464	1.693	7.089
Household Wealth index (ref: poor)	Medium	.617	.291	.034	1.854	1.048	3.281
	Rich	.816	.348	.019	2.261	1.143	4.475
ANC (ref: < 4)	≥ 4	.958	.258	.000	2.606	1.573	4.318
Transport access (ref: No)	Yes	.653	.260	.012	1.921	1.154	3.197
Media access (ref: No)	Yes	.682	.267	.011	1.979	1.173	3.339
Nagelkerke R Square	0.418						
Hosmer and Lemeshow Test	0.162						

*B* Coefficient, *S.E.* Standard error, *Sig. P* value, *Exp (B)* Odds ratio, *C.I. 95%* Confidence interval for odds ratio

### Factors associated with attitude in rural parts of Gesha Woreda, Kaffa, Southwest Ethiopia

Those mothers whose first pregnancy at age range 20–24 were 1.806 [95%CI: 1.086–3.005] times more likely to have a positive attitude regarding skilled maternal health care assistance compared to those mothers whose first pregnancy was at age interval 15–19. Women who had a planned pregnancy were 2.315 [95%CI: 1.367–3.922] times more likely to have a positive attitude towards skilled maternal health care assistance than an unplanned pregnancy. Mothers who had elementary education levels were 2.565 [95%CI: 1.472–4.470] times more likely to have a positive attitude in seeking skilled maternal health care assistance compared with those mothers who had no formal education. Married mothers were 1.762 [95%CI: 1.010–3.073] times higher chance to have a positive attitude in seeking skilled maternal care assistance compared to those mothers who were never married. Regarding age group, mothers in the age group 20–24, 25–29, and 35–49 were 4.989, 3.794, and 2.991 times more likely to have a positive attitude in seeking skilled maternal health care assistance than mothers in the age group 15–19 respectively. Husbands’ education level effect has a direct effect to have a positive attitude towards the service. Mothers whose husbands had elementary, and secondary &above education status were 2.542 and 3.360 times more likely to have a positive attitude toward getting skilled maternal care assistance compared to those mothers whose husbands had no formal education respectively. Mothers who had a household wealth index of medium and rich were 1.993 and 1.995 times more likely to have a positive attitude to service compared to their counterparts who were poor respectively. Women having greater than or equal to 4 ANC in their recent pregnancy were twice more likely to have a positive attitude toward skilled assistance as compared with those who visited antenatal care services less than 4 times were 2.570 [95%CI: 1.545–4.275]. Women who had access to transportation were 1.956 times more likely to have a positive attitude in seeking skilled maternal care services than those who had no transportation access 1.956 [95%CI: 1.168–3.275]. Mothers who had media access were 2.171 [95%CI: 1.278–3.687] times more likely to have a positive attitude towards skilled maternal care assistance compared to those mothers who had no access to media (Table 5).

**Table 5:** Responses of multivariable binary logistic regression result for attitude

Variables	Categories	B	S.E	Sig	Exp(B)	95% C.I.for EXP(B)	
						Lower	Upper
Age at first pregnancy (ref: 15–19)	20–24	.591	.260	.023	1.806	1.086	3.005
Plan for the pregnancy (ref: No)	Yes	.840	.269	.002	2.315	1.367	3.922
Maternal educational level (ref: No education)	Elementary	.942	.283	.001	2.565	1.472	4.470
	Secondary and above	.295	.360	.412	1.344	.663	2.722
Marital status (ref: never married)	Married/living together	.567	.284	.046	1.762	1.010	3.073
	Divorced/widowed	.604	.369	.101	1.830	.888	3.769
Maternal age (ref:15–19)	20–24	1.607	.351	.000	4.989	2.507	9.925
	25–29	1.334	.391	.001	3.794	1.763	8.165
	30–34	.483	.419	.250	1.620	.712	3.686
	35–49	1.095	.448	.015	2.991	1.242	7.200
Husband’s educational level (ref: No education)	Elementary	.933	.296	.002	2.542	1.424	4.540
	Secondary and above	1.212	.369	.001	3.360	1.630	6.924
Household Wealth index (ref: poor)	Medium	.690	.294	.019	1.993	1.120	3.549
	Rich	.691	.351	.049	1.995	1.002	3.973
ANC (ref: < 4)	≥ 4	.944	.260	.000	2.570	1.545	4.275
Transport access (ref: No)	Yes	.671	.263	.011	1.956	1.168	3.275
Media access (ref: No)	Yes	.775	.270	.004	2.171	1.278	3.687
Nagelkerke R Square	0.425						
Hosmer and Lemeshow Test	0.353						

*B* Coefficient, *S.E*. Standard error, *Sig. P* value, *Exp (B)* Odds ratio, *C.I. 95%* Confidence interval for odds ratio

### Factors associated with practice in rural parts of Gesha Woreda, Kaffa, Southwest Ethiopia

Age at first pregnancy had a significant effect on practicing skilled maternal care assistance. Mothers whose first pregnancy was in the age range 20–24 were 1.799 [95%CI: 1.799–3.065] times more likely to practice skilled maternal health care service compared to those mothers whose first pregnancy was at age interval 15–19. Mothers who had a planned pregnancy were 2.579 [95%CI: 1.475–4.512] times more likely in practicing skilled maternal health care assistance than an unplanned pregnancy. Mothers who had elementary education levels were 1.840 [95%CI: 1.840–5.960] times more likely to practice skilled maternal health care assistance compared with those mothers who had no formal education. Married mothers were 2.208 [95%CI: 1.222–3.989] times more likely in practicing skilled maternal care assistance compared to those mothers who were never married. Mothers in the age group 20–24, 25–29, and 35–49 were 5.870, 4.017and 3.472 times more likely to practice skilled maternal health care assistance than mothers in the age group 15–19 respectively. Mothers whose husbands had elementary, and secondary &above education status were 2.134 and 2.508 times more likely to practice skilled maternal care service compared to those mothers whose husbands had no formal education respectively. Mothers who had a household wealth index of medium and rich were 2.424 and 2.452 times more likely to practice skilled maternal care services compared to the poor. Women having greater than or equal to 4 ANC in their recent pregnancy were 2.689 times more likely to practice skilled maternal service as compared with those who visited antenatal care service less than 4 times 2.689 [95%CI: 1.571, 4.600]. Women who had access to transportation were 2.404 times more likely to practice skilled maternal care services than those who had no transportation access 2.404 [95%CI: 1.395–4.143]. Mothers who had media access were 2.715 [95%CI: 1.539, 4.787] times more likely to practice skilled maternal care service compared to those mothers who had no access to media (Table 6).

**Table 6:** Responses of multivariable binary logistic regression result for practice

Variables	Categories	B	S.E	Sig	Exp(B)	95% C.I.for EXP(B)	
						Lower	Upper
Age at first pregnancy (ref: 15–19)	20—24	.587	.272	.031	1.799	1.799	3.065
Plan for the pregnancy (ref: No)	Yes	.948	.285	.001	2.579	1.475	4.512
Pregnancy complication (ref:No)	Yes	.764	.277	.006	2.147	1.248	3.694
Maternal educational level (ref: No education)	Elementary	1.197	.300	.000	3.312	1.840	5.960
	Secondary and above	.508	.382	.183	1.661	.786	3.510
Marital status (ref: never married)	Married/living together	.792	.302	.009	2.208	1.222	3.989
	Divorced/widowed	.648	.389	.096	1.912	.891	4.102
Maternal age (ref:15–19)	20–24	1.770	.375	.000	5.870	2.814	12.247
	25–29	1.390	.414	.001	4.017	1.783	9.050
	30–34	.483	.455	.288	1.621	.665	3.951
	35–49	1.245	.472	.008	3.472	1.376	8.762
Husband’s educational level (ref: No education)	Elementary	.758	.309	.014	2.134	1.165	3.910
	Secondary and above	.920	.392	.019	2.508	1.162	5.413
Household Wealth index (ref: poor)	Medium	.886	.312	.004	2.424	1.317	4.464
	Rich	.897	.371	.016	2.452	1.184	5.077
ANC(ref: < 4)	≥ 4	.989	.274	.000	2.689	1.571	4.600
Transport access (ref: No)	Yes	.877	.278	.002	2.404	1.395	4.143
Media access (ref: No)	Yes	.999	.289	.001	2.715	1.539	4.787
Nagelkerke R Square	48.5						
Hosmer and Lemeshow Test	0.448						

*B* Coefficient, *S.E.* Standard error, *Sig. P* value, *Exp (B)* Odds ratio, *C.I. 95%* Confidence interval for odds ratio

## Discussion

One of the primary causes of maternal morbidity and mortality around the globe is a lack of access to maternal health services [39]. This study is one of the first to clarify a prior disagreement between the findings of many studies on knowledge, attitudes, and practice about the use of competent maternity care services. Leaving these tensions unresolved at the individual level might lead to policymakers and program designers missing the target point of intervention. Furthermore, this research paints a clear picture of the problem of low utilization of competent maternal care services as a continuum of care from pregnancy to postpartum. As a result, this study looked into the factors that influence knowledge, attitude, and practice in competent maternal care, focusing on a rural setting. For many years in Ethiopia, skilled mother care services such as prenatal care (ANC), delivery care (DC), and postnatal care (PNC) were mostly available to the rural population [40].

This study enlisted the participation of 424 women from rural areas of Gesha woreda. Knowledge, attitude, and practice of seeking competent assistance were 39.15 percent, 37.75 percent, and 34.67 percent, respectively, indicating that maternity care from skilled providers is underutilized. This research corresponds to a study conducted in Ethiopia's Oromia Region's West Shoa Zone [30]. But this study is low as compared with different studies in different countries [22, 25, 41]. The reasons for this discrepancy could be explained by differences in sample sizes, time intervals, and socioeconomic conditions in the different contexts.

From the influencing factors, age at first pregnancy, planned pregnancy, maternal education level, husband’s education level, maternal age, antenatal care service visits, transport access, and access to media was found to be associated with the knowledge, attitude, and practice in common. The household wealth index was also associated with attitude. Pregnancy complications, current marital status, and household wealth index also significantly affect the practice of seeking skilled maternal care assistance.

Women whose age at first pregnancy ranged from 20 to 24 were more likely to know, have a positive attitude, and practice skilled maternal care assistance such as antenatal care, delivery care, and postnatal care than adolescents and young mothers. Our findings were similar to those of research conducted in rural Bangladesh and Ghana [42, 43]. This could be because teenage girls are trying to hide their pregnancy and are embarrassed to be examined by health care providers.

Women who planned their pregnancy were more probable to know, have a positive attitude, and seek antenatal care, delivery care, and postnatal care than those women who had no plan for the pregnancy. This finding is consistent with research conducted in Ethiopia and the Democratic Republic of the Congo, which found that women who had unplanned pregnancies were less likely to give birth in a health facility than moms who had planned pregnancies [14, 30]. This could be because unwanted pregnancies are connected with discouragement and a lack of pregnancy experience, thus they are less inclined to seek maternity care.

Women’s education was significantly associated with skilled maternal care assistance (antenatal care service, delivery care service, and postnatal care service) utilization. Women with elementary education were more likely to know, have a positive attitude, and practice maternal health care services as compared to women with no education. The findings of this study were in line with other studies in Africa [8, 9, 11, 12, 44, 45] which highlighted that utilization of maternal health services increases consistently as the educational level increases. It is understood that education is likely to enhance women's autonomy and they are near to information and would have good knowledge [46]. The higher use of competent delivery services among well-educated women may be due to their level of understanding, which may lead to women being more worried about their health and disease and seeking appropriate healthcare services.

Lack of husband education had an influential effect on skilled maternal care service in the current study. However, other studies which were done in Bangladesh and Benishangul Gumuz of Ethiopia have disproved this study [47, 48]. The possible disparities might arise from the fact that husbands are more conservative towards their cultural practice of home delivery and women are not autonomous both in Bangladesh and Benishangul Gumuz of Ethiopia regardless of their educational status. This could indicate that education alone may not be enough to influence husbands' behavior unless significant effort is made to modify their attitudes.

Women who had experienced pregnancy complications were found to have a significant association with practicing skilled maternal health care services. Other research in Ethiopia's Oromia area corroborates this conclusion [21]. Mothers who were exposed to media on the importance of skilled maternal healthcare services were more likely to know, have a positive attitude and practice the services. This finding supports the study done in Ethiopia [13]. The number of antenatal care visits tended to increase the utilization of skilled.

delivery. Mothers who had visited the number of ANC services four times and above were more likely to know, have a positive attitude, and practice skilled delivery and postnatal care compared with those who visited less than four times. This finding is consistent with the studies in Ethiopia, Tanzania, South Sudan, and Nepal, respectively [18, 20, 28, 49]. This could be because antenatal care is an important intervention in bringing women into contact with the health system, making skilled birthing more accessible, and providing postnatal care. This means that having frequent antenatal care visits is critical for increasing the use of facility-based delivery services.

The results of this study showed that, to a great extent, women in the rich wealth index group had positive attitudes and practiced ANC, DC, and PNC. Studies done in Ethiopia and Bangladesh Came up with the same results as that in the current study [50, 51]. Even in locations where maternity healthcare is given free of charge, women must pay for transportation and other necessities. As a result, women who couldn't afford these costs found it difficult, if not impossible, to visit health care institutions.

The finding of this study showed that women who had access to transport were more likely to know, have a positive attitude, and practice antenatal care, postnatal care, and delivery care services than those who had no transport access. This finding is in line with a study conducted in Ghana, Kenya, and Malawi, which found that the availability of vehicles such as public transportation and taxis influenced pregnant women's decisions to seek maternity care [17]. This suggests that antenatal care consumption is inextricably linked to basic infrastructure.

## Conclusions

The study participants' knowledge, attitude, and practice toward skilled maternal health services are all low, according to the findings. Associated factors included age at first pregnancy, planned pregnancy, maternal education level, husband’s education level, maternal age, antenatal care service visits, transport access, and access to media were found to significantly affect the knowledge, attitude, and practice of the respondents in seeking skilled maternal care assistance in common. The household wealth index was also associated with attitude. Pregnancy complications, current marital status, and household wealth index also significantly affect the practice of seeking skilled maternal care assistance. As a result of the findings of this study, interventions to improve women's knowledge, attitude, and practice of skilled maternal health services in the study area are needed. Furthermore, unexpected pregnancy and a lack of mobility were both linked to a lack of use of maternal health services. As a result, the study recommends that integrated family planning and maternal healthcare services be revisited to help women with unexpected pregnancies access maternal healthcare services, as well as infrastructural improvements to improve access to maternal health services in rural areas of the country. Similarly, the use of competent delivery services is strongly linked to women's education. As a result, this research suggests that promoting equity among the excluded population is important.

### Limitations of the study

There are certain limitations to the research. The study employed a cross-sectional study design, which has significant methodological limitations when it comes to determining cause and effect links between factors. Because the information gathered from the participants may be influenced by social desirability due to recall bias, the study seeks to mitigate this by adding only women who had given birth in the previous 2 years.

## Data Availability

The datasets used in this study are available upon reasonable request from the corresponding author.

## Abbreviations

ANC: Antenatal care
DC: Delivery Care
KAP: Knowledge Attitude and Practice
PNC: Postnatal Care
SPSS: Statistical Package for Social Science
